# Management of glenohumeral osteoarthritis in the younger patient: A population level analysis of usual care delivery in a large health system

**DOI:** 10.1016/j.ocarto.2025.100705

**Published:** 2025-11-13

**Authors:** Daniel I. Rhon, Maggie E. Horn, Rebecca Milan, Steven Z. George

**Affiliations:** a Department of Rehabilitation Medicine, Brooke Army Medical Center, JBSA Fort Sam Houston, 3551 Roger Brooke Drive, TX, USA; b Department of Rehabilitation Medicine, Uniformed Services University, 4301 Jones Bridge Road, Bethesda, MD, USA; c Departments of Orthopaedic Surgery and Population Health Sciences, Duke Clinical Research Institute, Duke University, School of Medicine, 300 Morgan Street, Durham, NC, USA; d The Geneva Foundation, Tacoma, WA, USA; e Duke Clinical Research Institute, Duke University, School of Medicine, 300 Morgan Street, Durham, NC, USA

**Keywords:** Shoulder, Glenohumeral, Osteoarthritis, Younger patient, Military medicine

## Abstract

**Objective:**

To characterize healthcare utilization in a younger population with glenohumeral osteoarthritis.

**Methods:**

Adults seeking care for glenohumeral osteoarthritis between July 2013 and March 2019 were analyzed. Ambulatory visits, pharmacological, and non-pharmacological treatment, and surgical and radiological procedures, were compared by active-duty status and sex. The association between exercise therapy receipt and subsequent surgery was modeled using an adjusted logistic regression, reporting adjusted odds ratios (OR).

**Results:**

There were 21,369 individuals (29.4 ​% female; mean age 50.3 [standard deviation (SD) 9.9] years), with 24.1 ​% military patients. The mean number of ambulatory shoulder visits per patient was 9.4 (SD 12.5). For pharmacological care 26.2 ​% received an injection, 14.8 ​% received non-steroidal anti-inflammatory drugs, 13.4 ​% opioids, 11.8 ​% muscle relaxants, and 9.9 ​% other analgesics. Non-pharmacological care included 44.5 ​% receiving exercise therapy, 41.6 ​% physiotherapy, and 32.6 ​% manual therapy, while 54.0 ​% received none of these. Radiographs were obtained for 51.3 ​% and advanced imaging for 47.1 ​%. Arthroscopic surgery occurred in 18.9 ​% and arthroplasty in 0.2 ​%. Military patients received less pharmacological and more non-pharmacological treatment that non-military patients. Males had 1.5x the odds of surgery and higher pharmacological treatment use than females. Receipt of exercise therapy (adjusted OR ​= ​0.01; 95 ​% confidence interval 0.01, 0.02) significantly reduced the odds of arthroscopic surgery.

**Conclusion:**

Many patients received no interventions. For those receiving care, <50 ​% received guideline-recommended non-pharmacological care, including exercise therapy which significantly reduced the likelihood of surgery. Military patients had overall less pharmacological but more non-pharmacological treatment than non-military patients. A greater frequency of males had surgery. These findings indicate the need to further explore health care delivery for glenohumeral osteoarthritis in younger populations, highlighting potential guideline to practice gaps.

## 1 Introduction

The worldwide burden of osteoarthritis (OA) is substantial, affecting 528 million people globally (approximately 7 ​% of the world’s population) [1]. The estimated 2019 annual costs for managing a single individual with OA were estimated at $700-$15,600 (United States [U.S.] Dollars) [1]. The burden is driven primarily by disease prevalence in the knee, hand, and hip. Subsequently, less attention has been given to OA in other joints, despite their high prevalence. Up to 19 ​% of middle-aged and older adults show radiographic signs of glenohumeral OA [2], though many do not experience symptoms or seek care [3]. Of those who do, glenohumeral OA accounts for 5–17 ​% of shoulder-related visits. [2] As in the knee and hip, end-stage disease in the shoulder often leads to joint replacement. Between the years 2012–2017, total shoulder arthroplasty procedures increased substantially, from 9.5 cases per 100,000 to 12.5 cases per 100,000, with the greatest incidence occurring in patients >65 years of age [4]. This equated to an approximately 2-fold increase from approximately 50,000 procedures in 2012 to 103,000 procedures in 2017 [5]. The highest estimates project triple this number by the end of the year 2025 [6]. These numbers outpace projections of both knee and hip arthroplasty procedures during this same time [6].

Beyond hip and knee OA, glenohumeral OA is less well-researched and few clinical practice guidelines exist [[7], [8], [9]]. A review of 26 clinical practice guidelines for shoulder disorders found only 12 (46 ​%) were of high quality methodology as determined by the Appraisal of Guidelines for REsearch & Evaluation (AGREE) II checklist; but all with major limitations related to applicability [7]. Only five of these high-quality guidelines offered recommendations for glenohumeral OA. Acetaminophen and oral non-steroidal anti-inflammatory drugs (NSAID) were the pharmacological treatments recommended. The only two non-pharmacological treatments recommended were active exercises and physiotherapy. However, these also fell into the “may be recommended” category, highlighting the lack of conclusive evidence. For imaging procedures, MRI was not recommended, and for surgical procedures, arthroplasty was recommended for end-stage disease. Most recommendations were based on consensus, due to lack of reliable evidence [9]. Essentially, guidelines are few, of low quality, and rarely address younger patients. Real-world evidence is necessary [10], but lacking. Further research should be a priority as this younger age group is typically in the workforce longer and therefore early disability can carry greater long-term socioeconomic consequences. Characterizing current treatment patterns at the health system level can help determine what changes are needed to better align care with guideline recommendations.

Glenohumeral OA has been primarily studied in older populations >65 years of age. To our knowledge, no population-level cohort studies of younger patients with glenohumeral OA exist. It is unclear if the current evidence and treatment guidance derived primarily for an older population carries the same relevance for a younger population. Assessing current treatment utilization rates in younger patients may be the first step in understanding how care might differ from recommendations provided for older patients.

The purpose of this study was to identify gaps in guideline-adherent care in the management of glenohumeral OA for young adults (<65 years). The primary aim was to describe the real-world management of glenohumeral OA at the population level within a large US health network that consists primarily of a younger population. As sub aims, we wanted to compare differences 1) between sexes, due to known differences in their presentation, and 2) between military and non-military patients, due to the more physically demanding nature of military service and unique barriers to seeking care in this subgroup of the population [[11], [12], [13]]. The secondary aim was to determine whether receipt of exercise therapy, the only non-surgical and non-pharmacological intervention recommended by the guidelines for older adults [14], was associated with receipt of surgery within a year of initial glenohumeral OA diagnosis.

## 2 Methods

### 2.1 Study design and setting

This was a longitudinal cohort study using routinely collected health information to identify and track healthcare utilization from an initial shoulder visit, prospectively through a 1-year period of surveillance, regardless of the setting where initial care began (primary, specialty, or emergency care). In this cohort, the longer the follow-up the greater the reduction the sample size based on requirements to be eligible to receive care in this health system. For this reason, and to focus on initial care pathways, we chose to focus on a cohort with one-year follow-up. Cases were sourced from Tricare, one of the largest healthcare networks in the US, providing care for approximately 9.5 million beneficiaries around the world. Health plan beneficiaries include active and retired service members and their dependents. This military cohort is ideal to answer this question, as they are younger and with known incidence rates of osteoarthritis approximately double that of the general population [15]. This single-payer government system is optimal for answering this question as very few individuals receive outside care that cannot be tracked. The study received ethics approval from the Institutional Review Board at the US Army Medical Center of Excellence The REporting of studies Conducted using Observational Routinely-collected Data [16] extension of the Strengthening the Reporting of OBservational studies in Epidemiology checklist was used to guide reporting.

### 2.2 Data source

The U.S. Military Health System Data Repository (MDR) was used to source the data [17]. This repository includes data from electronic medical records sourced from healthcare encounters taking place in the 32 military hospitals and 480+ ambulatory clinics around the world, in addition to claims data from care outsourced to private sector network clinics. The MDR receives feeds from multiple data sources which are continuously validated for at least ninety days. Data includes all ambulatory visits, radiology procedures, and medication prescriptions. Person-level data were abstracted by an analyst and provided in raw form, de-identified to the study team.

### 2.3 Variables

#### 2.3.1 Cohort selection

**Fig. 1:**
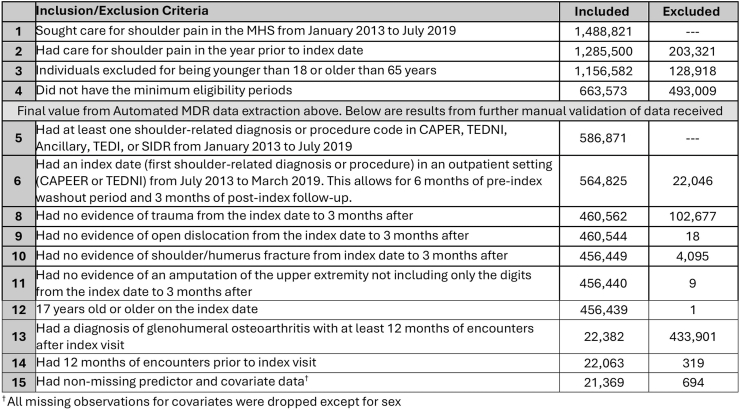
Cohort selection flow.

Patients were identified based on ambulatory visits coded with a glenohumeral OA International Statistical Classification of Diseases and Related Health Problems (ICD) code, 9th (715.11, 715.21, 715.31, 715.91) or 10th edition (M19.90, M19.91, M19.92, M19.01x, M19.11x, M19.21x). The care epoch spanned July 2013 to March 2019. For inclusion in the cohort, eligibility to receive care in this health system was required for a full 12-month lookback period. We then required 12 months of eligibility after the index date. This was part of a larger cohort assessing general shoulder pathology in this health system. For that reason, any patients with an upper extremity amputation, open glenohumeral dislocation, or fractures of the shoulder and humerus during the one-year prior had been excluded. Patients were allowed to have other shoulder disorder diagnoses in addition to that of glenohumeral OA. We only included adults from ages 18 to 65. While this excludes an older demographic where prevalence of glenohumeral OA is expected to be much higher, it allows a focus on a younger, more active population. The cohort selection flow is shown in Fig. 1. The MDR also categorizes all patients by beneficiary status, allowing separation of military service members on active duty from everyone else. It is worth nothing that our cohort represents two age subgroups, both considered younger patients with glenohumeral OA. The military cohort had a median age of 40 years and the non-military cohort a median age of 55 years.

#### 2.3.2 Care selection and source

We categorized all shoulder care during the three months following the index date, as well as the number of shoulder-related ambulatory visits 12 months prior. We wanted to capture initial care decisions, focusing on care delivery in this initial 3-month period. The exception was surgical procedures, for which we allowed for an entire year, as this was more of an outcome rather than an initial care decision. We characterized the type of shoulder-related care using Current Procedural Terminology and Healthcare Common Procedure Coding System codes linked to shoulder-related ambulatory visits (supplementary appendix). This means that after identifying the original OA diagnosis, we counted any care for the shoulder, regardless of diagnostic label. For all follow-on care, we included events from both military and private sector network clinics. We also included shoulder-related radiology records from the Ancillary file and prescription data from the Pharmacy Data Transaction Service file using American Hospital Formulary Service therapeutic class codes, all sourced from the MDR.

#### 2.3.3 Pain-related comorbidities

We identified pain-related comorbidities based on ambulatory visits where a relevant ICD diagnosis was rendered by a licensed medical professional, as has been described previously [17,18]. A care-seeking event with the comorbidity diagnosis rendered in the six months prior to the index date was required to classify the comorbidity for each patient. Conditions included insomnia, sleep apnea, anxiety, depression, post-traumatic stress disorder, cardiovascular disease, metabolic syndromes, and substance abuse disorders, as well as any additional sleep and mental health disorders (specific codes in the supplementary appendix).

#### 2.3.4 Care variable classification

Similar to our prior work [19], we classified care into three main groups: pharmacological, non-pharmacological, and procedures (imaging and surgical). The number of prescriptions and treatments per patient and a binary indicator (yes/no) of whether the patient received the specific treatment were calculated. This was based on whether treatment procedure code was billed during a patient visit, or a medication prescription filled by a patient, based on data in electronic medical records for visits in the 3 months after the index date. Pharmacological treatments included NSAIDs, opioids, muscle relaxers, analgesics, and drugs delivered by injection to the glenohumeral joint (glucocorticoid and viscosupplementation). These were identified using the American Hospital Formulary Service and Healthcare Common Procedure Coding System codes, using previously described methodology [20]. The other analgesics category included drugs such as acetaminophen and lidocaine patches. Non-pharmacological treatments included whether they had at least one consultation with a physiotherapist, and whether they received at least one visit for exercise therapy, therapeutic modalities (considered passive), manual therapy, and/or acupuncture. Therapeutic modalities would include treatments such as therapeutic ultrasound, electrical nerve stimulation, hot and cold therapy, and light therapy. Imaging procedures included radiographs, MRI, arthrograms, and CT scans. The latter three categories were also combined into a single category classified as “advanced imaging”. Surgical procedures were further categorized into shoulder arthroscopy and shoulder arthroplasty. All codes are listed in the supplementary appendix.

### 2.4 Statistical analysis

The number of prescriptions and type of treatment each patient received was calculated. To determine receipt of treatment or prescriptions, we created binary variables (yes/no). If a patient had 1 or more visits, where the visit billed a Current Procedural Terminology code for the treatment or placed a prescription order that was filled for the medication class codes within the first three months following the index date, it was coded a “yes”. If no codes were found for a treatment or medication class, then it was assumed to not have occurred and coded a “no”. Descriptive statistics, including mean and standard deviations, summarized the distribution of care per patient. Healthcare utilization was also compared between military patients and all others. Chi-square and Wilcoxon rank sum tests were used to test the difference in care receipt between clinician types and settings. Given the large sample size of this study, a small non-clinically important difference can be claimed as statistically significant [21]. Therefore, we prioritized the focus on clinical relevance and did not perform inferential statistical testing when comparing groups. Finally, we modeled the association between receipt of exercise therapy and surgery occurrence within 1-year after diagnosis using a generalized estimating equations logistic regression to best understand the population-averaged effects. We used robust standard errors, a logit link function, and an independent correlation structure to account for repeated measures occurring at the individual level. While data were clustered at the facility level, the research question was focused on the association of exercise with surgery for the entire cohort, not across facilities. The model was also adjusted for sex, age, and military (active duty) status. We used multiple imputation by chained equations to account for the large amount of missing sex observations (>10 ​%). Sex and age are key determinants in OA and well-known to influence healthcare utilization [22,23], and military populations are at much higher risk for developing OA and OA-related disability [15,24]. We reported adjusted odds ratios with 95 ​% confidence intervals. We prespecified our plan to model each of the surgery outcomes separately, with one regression model for arthroscopic surgery and another one for arthroplasty. However, we ended up only modeling for arthroscopic surgery because there were such few cases of arthroplasty in this cohort.

## 3 Results

There were 21,369 patients seeking care for glenohumeral OA that met inclusion criteria during the surveillance period (29.4 ​% female, mean age of 50.3 [standard deviation 9.9], 24.1 ​% on active duty; Fig. 1). There were 12.4 ​% (*N* ​= ​2643) of eligible individuals with one visit to receive the diagnosis, and then no additional shoulder-related visits in the following year. Most patients were seen in civilian network clinics (68.7 ​%). However, within the subgroup that were on active duty, the majority received the initial diagnosis from a military clinic (60.3 ​%). Further demographic details based on active-duty status are available in Table 1 and based on sex in the supplementary appendix (sex was missing for 12.7 ​% of the cohort, limiting the accuracy of sex-based comparisons).

**Table 1:** Demographic characteristics of glenohumeral OA cohort by active-duty status.

	Total *N* ​= ​21,369	Active Duty *N* ​= ​5158 (24.1 ​%)	Not Active Duty *N* ​= ​16,211 (75.9 ​%)
**Index clinic setting – (N%)**			
Private sector	14,678 (68.7 ​%)	2048 (39.7 ​%)	12,630 (77.9 ​%)
Military treatment facility	6691 (31.3 ​%)	3110 (60.3 ​%)	3581 (22.1 ​%)
**Sex – (N%)**			
Female	6286 (29.4 ​%)	364 (7.1 ​%)	5922 (36.5 ​%)
Male	12,374 (57.9 ​%)	4216 (81.7 ​%)	8158 (50.3 ​%)
Missing	2709 (12.7 ​%)	578 (11.2 ​%)	2131 (13.2 ​%)
**Age**			
Mean (SD)	50.3 (9.9)	40.1 (8.1)	53.6 (8.1)
Median (Q1, Q3)	52.0 (44.0, 58.0)	40.0 (35.0, 46.0)	55.0 (49.0, 60.0)
**Rank status**^a^**– (N%)**			
Enlisted	16,133 (75.6 ​%)	3508 (68.0 ​%)	12,625 (77.9 ​%)
Officer	5193 (24.3 ​%)	1642 (31.8 ​%)	3551 (21.9 ​%)
Cadet	8 (0.04 ​%)	8 (0.2 ​%)	–
Other/Unknown	20 (0.1 ​%)	–	20 (0.1 ​%)
**Service branch**^a^**– (N%)**			
Army	8986 (42.1 ​%)	2457 (47.6 ​%)	6529 (40.3 ​%)
Air force	6060 (28.4 ​%)	1084 (21.0 ​%)	4976 (30.7 ​%)
Navy	4118 (19.3 ​%)	848 (16.4 ​%)	3270 (20.2 ​%)
Marines	1515 (7.1 ​%)	446 (8.7 ​%)	1069 (6.6 ​%)
Coast guard	578 (2.7 ​%)	267 (5.2 ​%)	311 (1.9 ​%)
Other/Unknown	112 (0.5 ​%)	56 (1.1 ​%)	56 (0.4 ​%)
**Shoulder encounters per patient during 12 months before index visit for GH OA**			
Mean (SD)	1.9 (3.0)	2.4 (3.4)	1.8 (2.9)
Median (Q1, Q3)	1 (0, 2)	1 (0, 3)	1 (0, 2)
Range	0–58	0–41	0–58
**Shoulder encounters per patient during 12 months after index visit for GH OA**			
Mean (SD)	9.4 (12.5)	10.7 (14.1)	9.1 (12.0)
Median (Q1, Q3)	4 (2, 13)	5 (2, 14)	4 (1, 12)
Range	(0–123)	(0–112)	(0–123)
**Comorbidities at index visit – N (%)**			
Insomnia	1383 (6.5 ​%)	427 (8.3 ​%)	956 (5.9 ​%)
Sleep apnea	3067 (14.4 ​%)	906 (17.6 ​%)	2161 (13.3 ​%)
Other sleep disorders	1119 (5.2 ​%)	512 (9.9 ​%)	607 (3.7 ​%)
Depression	1700 (8.0 ​%)	282 (5.5 ​%)	1418 (8.7 ​%)
Anxiety	1645 (7.8 ​%)	368 (7.1 ​%)	1277 (11.3 ​%)
PTSD	693 (3.2 ​%)	291 (5.6 ​%)	402 (2.5 ​%)
Other mental health disorders	1622 (7.6 ​%)	520 (10.1 ​%)	1102 (6.8 ​%)
Cardiovascular disease	7295 (34.1 ​%)	707 (13.7 ​%)	6588 (40.6 ​%)
Metabolic syndromes	8188 (38.3 ​%)	881 (17.1 ​%)	7307 (45.1 ​%)
Substance abuse disorders	1551 (7.3 ​%)	407 (7.9 ​%)	1144 (7.1 ​%)

Abbreviations: SD= Standard Deviation; Q1 ​= ​1st quartile; Q3 ​= ​3rd quartile.

Comorbidities represent a medical encounter with the diagnosis for this condition rendered, during the six months prior to the index date. Active duty refers to individuals actively serving in the military full time, in comparison to any individual not in military service (either a dependent or someone separated or retired from military service).

a Represents status of the sponsor (active duty or retired service member) if not an individual on active duty.

### 3.1 Pharmacological care

The most common pharmacological treatment was drugs delivered by injections to the glenohumeral joint, received by 26.2 ​% of the cohort, with a median (Interquartile Range - IQR) of sixteen (0,64) days to the first injection. Within this group, the majority were glucocorticoid injections (26.1 ​% of the cohort). The next most common pharmacological treatment was non-steroidal anti-inflammatory drugs (NSAIDS), with prescriptions filled by 14.8 ​% of the cohort at a median of 19 days to the first fill. Opioid and muscle relaxer use came closely behind, with prescriptions filled by 13.4 ​% and 11.8 ​% of the cohort, respectively. Patients who used opioids filled a median of five prescriptions, the first a median of 29 days from the date of initial diagnosis (Table 2). Other analgesic prescriptions were filled by 9.9 ​% of the cohort.

**Table 2:** Pharmacological Therapy by active duty status.

	Total *N* ​= ​21,369	Active Duty *N* ​= ​5158 (24.1 ​%)	Not Active Duty *N* ​= ​16,211 (75.9 ​%)
**NSAIDs – (N%)**	3154 (14.8 ​%)	628 (12.2 ​%)	2526 (15.6 ​%)
Mean unique fills (SD)	8.5 (5.4)	8.2 (5.3)	8.6 (5.4)
Median unique fills (Q1, Q3)	8 (4, 12)	7 (4, 11)	8 (4, 12)
Range	1–36	1–36	1–30
Median (Q1, Q3) days to first fill	19 (1, 60)	22 (4, 65)	18 (1, 58)
**Opioids – (N%)**	2860 (13.4 ​%)	572 (11.1 ​%)	2288 (14.1 ​%)
Mean unique fills (SD)	8.1 (8.9)	7.8 (8.9)	8.1 (9.0)
Median unique fills (Q1, Q3)	5 (2, 10)	5 (2, 9)	5 (2, 10)
Range	1–86	1–61	1–86
Median (Q1, Q3) days to first fill	29 (7, 85)	32 (9, 95.5)	28 (6, 83.5)
**Analgesics – (N%)**	2124 (9.9 ​%)	423 (8.2 ​%)	1701 (10.5 ​%)
Mean unique fills (SD)	2.5 (2.1)	2.6 (2.4)	2.4 (2.0)
Median unique fills (Q1, Q3)	2 (1, 3)	2 (1, 3)	2 (1, 3)
Range	1–26	1–26	1–21
Median (Q1, Q3) days to first fill	53 (0, 154)	48 (0, 147)	55 (1, 157)
**Muscle relaxers – (N%)**	2511 (11.8 ​%)	493 (9.6 ​%)	2018 (12.4 ​%)
Mean unique fills (SD)	3.9 (3.6)	3.7 (3.8)	3.9 (3.6)
Median unique fills (Q1, Q3)	3 (1, 5)	2 (1, 5)	3 (1, 5)
Range	1–33	1–29	1–33
Median (Q1, Q3) days to first fill	52 (8, 139)	72 (19, 155)	48.5 (6, 133)
**Pharmacological injections – (N%)**	7891 (36.9 ​%)	1831 (35.5 ​%)	6060 (37.4 ​%)
Mean number of injections (SD)	1.5 (0.9)	1.4 (0.8)	1.5 (0.9)
Median number of injections (Q1, Q3)	1 (1, 2)	1 (1, 2)	1 (1,2)
Range	(1–18)	(1–12)	(1–18)
Median (Q1, Q3) days to first	14 (0, 56)	14 (0, 60)	14 (0, 55)
**Glucocorticoid injections – (N%)**	5577 (26.1 ​%)	1016 (19.7 ​%)	4561 (28.1 ​%)
Mean number of injections (SD)	1.4 (0.7)	1.3 (0.7)	1.4 (0.7)
Median number of injections (Q1, Q3)	1 (1, 2)	1 (1, 1)	1 (1, 2)
Range	1–14	1–8	1–14
Median (Q1, Q3) days to first	16 (0, 64)	18 (0, 68.5)	15 (0, 63)
**Viscosupplementation injections – (N%)**	57 (0.3 ​%)	12 (0.2 ​%)	45 (0.3 ​%)
Mean number of injections (SD)	1.3 (0.7)	1.3 (0.5)	1.3 (0.7)
Median number of injections (Q1, Q3)	1 (1, 1)	1 (1, 1.5)	1 (1, 1)
Range	1–4	1–2	1–4
Median (Q1, Q3) days to first	56 (14, 136)	102 (49, 127.5)	51 (13, 138)

Abbreviations: SD= Standard Deviation; Q1 ​= ​1st quartile; Q3 ​= ​3rd quartile.

Active duty refers to individuals actively serving in the military full time, in comparison to any individual not in military service (either a dependent or someone separated or retired from military service).

#### 3.1.1 Pharmacological care secondary aims

Pharmacological use was mostly comparable to all other individuals for all pharmacological use (Table 2). Pharmacological use also did not vary substantially by sex across any of the drug categories (see Table SA2 in Supplementary Appendix).

### 3.2 Non-pharmacological care

Less than half of the cohort used non-pharmacological care (Table 3), as 44.5 ​% of patients received exercise therapy, 41.6 ​% were evaluated by a physiotherapist, and 32.6 ​% received manual therapy. Therapeutic passive modalities were used in 23.3 ​% of cases. Most patients received a multimodal approach that included all four types of non-pharmacological care, with <5 ​% of patients receiving either of these interventions in isolation (**see** Figure SA1 in supplementary appendix). Acupuncture was used for 0.3 ​% of cases. The median days to the first treatment within all categories was >30 days (Table 3).

**Table 3:** Non-pharmacological care by military status.^a^

	Total *N* ​= ​21,369	Active Duty *N* ​= ​5158 (24.1 ​%)	Not Active Duty *N* ​= ​16,211 (75.9 ​%)
**Physical therapy – N (%)**	8895 (41.6 ​%)	2549 (49.4 ​%)	6346 (39.1 ​%)
Mean (SD)	3.9 (7.1)	6.8 (10.0)	2.7 (5.0)
Median (Q1, Q3)	1 (1, 3)	2 (1, 8)	1 (1, 2)
Range	(1–107)	(1–107)	(1–74)
Median (Q1, Q3) days to first	35 (14, 85)	34 (11, 91)	35 (15, 84)
**Therapeutic modalities – N (%)**	4979 (23.3 ​%)	1440 (27.9 ​%)	3539 (21.8 ​%)
Mean (SD)	8.9 (9.7)	8.6 (9.8)	9.0 (9.6)
Median (Q1, Q3)	6 (2, 12)	5 (2, 11)	6 (2, 12)
Range	(1–83)	(1–83)	(1–73)
Median (Q1, Q3) days to first	42 (16, 101)	52 (16, 120.5)	40 (16, 93)
**Exercise therapy – N (%)**	9512 (44.5 ​%)	2599 (50.4 ​%)	6913 (45.8 ​%)
Mean (SD)	11.0 (11.3)	11.2 (12.2)	11.0 (11.0)
Median (Q1, Q3)	7 (3, 15)	7 (3, 15)	7 (3, 15)
Range	(1–106)	(1–105)	(1–106)
Median (Q1, Q3) days to first	34 (13, 84)	34 (11, 90)	34 (14, 82)
**Manual therapy – N (%)**	6958 (32.6 ​%)	1742 (33.8 ​%)	5216 (32.2 ​%)
Mean (SD)	9.8 (10.4)	9.0 (10.7)	10.0 (10.3)
Median (Q1, Q3)	6 (2, 13)	5 (2, 12)	6 (3, 14)
Range	(1–109)	(1–84)	(1–109)
Median (Q1, Q3) days to first	44 (17, 98)	55 (19, 120)	42 (17, 92)
**Acupuncture – N (%)**	56 (0.3 ​%)	37 (1.0 ​%)	19 (0.1 ​%)
Mean (SD)	2.7 (5.5)	3.1 (6.7)	1.8 (1.2)
Median (Q1, Q3)	1 (1, 2)	1 (1, 2)	1 (1, 3)
Range	1–41	1–41	1–4
Median (Q1, Q3) days to first	97 (17, 197)	111 (27, 227)	77 (6, 190)

Abbreviations: SD= Standard Deviation; Q1 ​= ​1st quartile; Q3 ​= ​3rd quartile.

Therapeutic modalities include treatment such as therapeutic ultrasound, electrical stimulation, hot or cold therapy, and laser or light therapy.

a Values represent individuals who received the procedure at least once.

#### 3.2.1 Non-pharmacological care secondary aims

The use of non-pharmacological care was slightly higher for those on active duty compared to all other individuals. There were no differences of receipt of non-pharmacological care by sex across all categories (**see** Table SA3 in supplementary appendix).

### 3.3 Imaging and surgical procedures

Radiographs of the glenohumeral joint were ordered by 51.3 ​% of the cohort, and advanced imaging procedures by 47.1 ​% of the cohort. For those who had imaging, median days to both radiographs and advanced imaging was zero days, indicating most occurred the same day as the initial visit. Shoulder arthroscopic surgery took place in 18.9 ​% of the cohort, with a slightly higher frequency of those on active duty (23.6 ​%) receiving the procedure compared to other beneficiaries (17.3 ​%). The mean number of days from the initial diagnosis to arthroscopic surgery was 91.2 (SD 92.1). There were only 41 total cases of shoulder arthroplasty (0.2 ​%; Table 4).

**Table 4:** Imaging and surgical procedures by active-duty status.

	Total *N* ​= ​21,369	Active Duty *N* ​= ​5158 (24.1 ​%)	Not Active Duty *N* ​= ​16,211 (75.9 ​%)
**Radiographs – N (%)**	10,967 (51.3 ​%)	1873 (36.3 ​%)	9094 (56.1 ​%)
Mean (SD)	1.9 (1.4)	1.8 (1.4)	1.9 (1.4)
Median (Q1, Q3)	1 (1, 2)	1 (1, 2)	2 (1, 2)
Range	(1–19)	(1–12)	(1–19)
Median (Q1, Q3) days to first procedure	0 (0, 17)	0 (0, 45)	0 (0, 12)
**Advanced imaging (MRIs, arthrograms, CT scans) - N (%)**^a^	10,068 (47.1 ​%)	2257 (43.8 ​%)	7811 (48.2 ​%)
Mean (SD)	1.8 (0.9)	1.9 (0.9)	1.8 (0.9)
Median (Q1, Q3)	2 (1, 2)	2 (1,2)	2 (1, 2)
Range	(1–10)	(1–8)	(1–10)
Median (Q1, Q3) days to first procedure	0 (0, 22)	0 (0, 12)	0 (0, 26)
**Any imaging procedure – N (%)**	15,313 (71.7 ​%)	2950 (57.2 ​%)	12,363 (76.3 ​%)
Mean (SD)	2.5 (1.8)	2.5 (1.8)	2.5 (1.8)
Median (Q1, Q3)	2 (1, 3)	2 (1, 3)	2 (1, 3)
Range	1–27	(1–16)	(1–27)
Median (Q1, Q3) days to first procedure	0 (0, 0)	0 (0, 0)	0 (0, 0)
**Surgery – N (%)**			
Shoulder arthroscopy	4031 (18.9 ​%)	1219 (23.6 ​%)	2812 (17.3 ​%)
- Mean (SD) days to arthroscopy	91.2 (92.1)	96.8 (91.0)	88.7 (92.5)
Shoulder arthroplasty	41 (0.2 ​%)	6 (0.1 ​%)	35 (0.2 ​%)
- Mean (SD) days to arthroplasty	130.4 (98.2)	142 (157.1)	128.5 (87.7)
Other surgical shoulder procedure^b^	1177 (5.5 ​%)	388 (7.5 ​%)	789 (4.9 ​%)
- Mean (SD) days to other procedure	100.5 (96.3)	103.6 (93.6)	99.0 (97.7)

Abbreviations: SD= Standard Deviation; Q1 ​= ​1st quartile; Q3 ​= ​3rd quartile; MRI ​= ​magnetic resonance imaging; CT ​= ​computed tomography.

Active duty refers to individuals actively serving in the military full time, in comparison to any individual not in military service (either a dependent or someone separated or retired from military service).

a The majority of individuals who received advanced imaging procedures had an MRI (98.2 ​% total, 98.9 ​% active-duty, 98.0 ​% non-active duty).

b Examples of other shoulder procedures included capsular release, open bankart repair, biceps tenodesis.

#### 3.3.1 Procedure-related secondary aims

With radiographs, more non-military individuals received a radiograph than active-duty military patients (56.1 ​% versus 36.3 ​%). The frequency of individuals receiving advanced imaging was similar between active-duty military and all other beneficiaries (Table 4). Imaging procedures of all types had similar frequencies by sex (**see** Table SA4 in supplementary appendix). More male patients underwent shoulder arthroscopic surgery than female (20.9 ​% versus 15.8 ​%; Table 4). Patients who received exercise therapy had significantly (99 ​%) lower odds of undergoing arthroscopic surgery (adjusted odds ratio ​= ​0.01; 95 ​% Confidence Interval 0.01, 0.02; *P* ​< ​0.001; Table 5).

**Table 5:** Factors associated with receiving arthroscopic surgery within one year of initial diagnosis (*N* ​= ​21,369).

Variable	Adjusted Odds Ratio	95 ​% Confidence Intervals	P Value
Age	0.98	0.98, 0.98	**<0.01**
Male sex	1.50	1.43, 1.58	**<0.01**
Active duty^a^	0.65	0.61, 0.69	**<0.01**
Receipt of exercise therapy	0.01	0.01, 0.02	**<0.001**

a Active duty refers to individuals actively serving in the military full time, in comparison to any individual not in military service (either a dependent or someone separated or retired from military service).

## 4 Discussion

Care patterns for this cohort revealed rates of arthroscopic surgery and diagnostic imaging not in line with clinical practice guideline recommendations, and room for improvement in the use of recommended non-surgical treatments. In general, very few treatments monitored for this study were rendered. For those receiving treatment, pharmacological care was common. Glucocorticoid injections were the most common pharmacological treatment, and approximately 13 ​% of the cohort received opioids. Non-pharmacological treatment of exercise or physiotherapy were used in less than half of this cohort. Imaging was common, with over 70 ​% of the cohort receiving at least one imaging procedure. Approximately 1 in 5 patients underwent an arthroscopic shoulder procedure, and essentially no one had an arthroplasty (0.2 ​%). Military personnel received fewer pharmacological treatments and imaging procedures, but higher non-pharmacological treatments and surgical procedures compared to everyone else. Receipt of pharmacological or non-pharmacological treatment did not differ by sex. More males underwent arthroscopic surgery than females. Receipt of exercise therapy was associated with 99 ​% lower odds of having surgery.

The utilization rates reported in this study reflect care for a much younger population than is traditionally assessed with glenohumeral OA. Management of glenohumeral OA in young patients (<60 years) has reported to be challenging [26], with the large bulk of the current literature focusing on case series and small cohorts of individuals receiving surgery. Research is needed to further study non-surgical and non-pharmacologic treatments in younger patients as there may be high potential for benefit from these interventions. For example, exercise therapy could have an even greater effect in this younger population, during potentially earlier stages of the disease [25], but was used in just under half of the cohort. Our results suggest a large and substantial reduction in risk of shoulder surgery when an individual receives exercise therapy as part of their treatment.

OA is one of the top two conditions most commonly responsible for medical separations from military service, even during times of peace [27]. In this cohort, a higher frequency of military personnel received arthroscopic surgery than non-military personnel (23.6 ​% vs 17.3 ​%). The low rates of arthroplasty are not surprising, considering this is usually a final treatment option, and generally not optimal for younger patients. Their use of pharmacological care was lower across all categories of pain medication, compared to non-military personnel. Although military personnel had lower rates of chronic disease compared to the older non-military subgroup (see Table 1), the prevalence of these conditions merits considerations when determining treatment strategies for this demographic, who often also have higher rates of post-traumatic OA [28]. The identified care patterns suggest different treatment strategies take place with younger patients, establishing a need for trials of non-surgical care in younger patients to better elucidate effective treatment strategies.

Very few cohort studies exist assessing treatment patterns for glenohumeral OA at health system levels and no other studies we are aware of have assessed these trends in a younger population. Thus, comparisons are difficult with existing literature but there are some studies to consider when interpreting these findings. For example, a prospective study of 5380 patients seeking emergency or urgent care with a primary diagnosis of glenohumeral OA found that 30 ​% received opioid prescriptions [29]. That was higher than our cohort, which received opioids at a rate of 13.9 ​% (11.7 ​% of active-duty; 14.7 ​% non-active-duty); however, our cohort assessed care beyond emergency/urgent care settings. In our cohort, opioids were used at almost the same rate as NSAIDs, despite recommendations for stepped care approaches, such as the World Health Organization analgesic ladder and the US Centers for Disease Control and Prevention guidelines [29,30] A systematic review of clinical practice guidelines for glenohumeral OA recommended NSAIDs and acetaminophen, but opioid use recommendations were conditional [7]. The median days from initial diagnosis to first NSAID fill was 19 compared to 29 for the first opioid fill. Even if the same individuals were receiving both medications, the 10 days between prescriptions would not be long enough to assess the benefit of NSAIDs. These stepped care approaches are recommended because opioids show similar or decreased effectiveness for chronic pain conditions than NSAIDS, but come with much higher risks (e.g., dependency, misuse).^30^The same guidelines for glenohumeral OA recommended physiotherapy and exercise therapy [7], which were used by less than half of our cohort (41.6 ​% and 44.5 ​%, respectively). The guidelines also recommend only plain radiographs [7], yet almost 50 ​% of our cohort received an MRI. Finally, nearly one in five patients received arthroscopic surgery, despite the lack of evidence for its use. A high-quality trial found no benefit of arthroscopic surgery for subacromial pain, to include cases caused from degenerative diseases such as OA [31]. No trials exist comparing arthroscopic surgery to placebo or non-surgical controls in patients with glenohumeral OA, regardless of age. These findings of high surgical rates in this patient population highlight potential gaps in research to practice, that need further investigation. They also emphasize a need for re-assessment of current practice guidelines for relevance to a younger population.

These findings may have limited generalizability. The cohort consisted of younger adults (<65 years), included about 25 ​% military personnel—who are known to have higher OA rates than civilians [15]—and was drawn from a single-payer government health system, which differs from the predominantly private, insurance-based systems in the U.S. Although most care occurred in civilian hospitals, these factors may limit applicability to older populations and other healthcare settings. However, most (∼70 ​%) of the care initiated in civilian network hospital settings also manage the general civilian population. These trends reflect their treatment pathways to a greater degree than care delivered in military clinics. Despite this high volume, only a marginal amount of research has focused on younger adults with glenohumeral OA.

### 4.1 Strengths and limitations

The strength of this study is the size of the cohort, essentially at the population-level (rather than just a sample) within an entire health system. This lends to increased pragmatism and generalizability of our results within this setting. As a government socialized medical system, capture of actual care received is extremely accurate, in contrast to civilian health systems in the U.S., which are mostly 3rd party payer insurance based. The limitations include the chronic nature of OA which brings unique challenges to surveilling its healthcare utilization, especially when using data not originally created to answer this question. First, knowing the classification or stage of disease may have increased the clinical relevance Second, patients could have other shoulder diagnoses in addition to glenohumeral OA, and some may have sought care earlier under different or nonspecific diagnostic labels. Secondary osteoarthritis is more common in younger adults [32,33]. These factors introduce heterogeneity and may affect how healthcare utilization and timing are measured, although they also reflect real-world complexity and improve generalizability. In addition, the cohort from where this diagnostic subgroup was derived had excluded any cases with trauma (e.g., fractures, open dislocation) occurring in the year prior to the index date. While these traumatic injuries can increase the risk for OA, most traumatic OA takes longer than a year to manifest [34], and no cases with traumatic injuries prior to the one-year window were excluded. The majority of OA has historically been considered idiopathic, but the true rate is unknown, especially in a younger population where a high prevalence of shoulder injuries may be a precursor for the disease. Due to the observational nature of our study, treatment allocation was not random. Patients who received exercise therapy may differ systematically from those who did not (e.g., in disease severity), introducing potential selection bias despite adjustment for measured confounders. Finally, for non-pharmacological care we calculated the type of clinicians seen (physiotherapist) and then specific interventions which often fall under the umbrella of physiotherapy (e.g., exercise, manual therapy). While this may provide some challenges with interpretation, this was because there are many interventions that fall under the label of physiotherapy, and physiotherapists are not the only profession whose scope of practice includes these treatments.

### 4.2 Clinical implications and future research

These findings highlight areas for which care delivery could potentially be improved. For example, improving delivery of guideline-recommended exercise therapy. This includes assessing implementation barriers and other reasons for the discrepancy between practice and guideline recommendations. This is especially true for younger patients who may respond favorably to exercise, limiting the need for invasive treatment approaches. There could also be opportunities for de-implementation initiatives across the health system, related to advanced imaging, surgical procedures, and opioid prescription practices. Finally, future studies are also necessary to better understand if guideline adaptations are necessary if younger patients respond differently to treatments.

## 5 Conclusion

In our cohort of younger patients with glenohumeral OA (median age 55), fewer than half received guideline-recommended non-pharmacological care. In contrast, nearly half underwent advanced imaging (mostly MRI) and a substantial proportion received arthroscopic surgery, both of which are not supported by current guidelines. Those who did receive exercise therapy had 99 ​% lower odds of receiving arthroscopic surgery. Military patients had a lower rate of pharmacological treatment, but higher rate of non-pharmacological treatment and surgery compared to non-military patients. A higher frequency of military patients also received advanced imaging procedures. No differences by sex were observed, except a higher rate of males received surgery than females. These findings highlight significant gaps in adherence to evidence-based recommendations. Efforts should focus on understanding the reasons for poor adherence and developing strategies to improve implementation in clinical practice.

## Disclaimer

The views expressed in this manuscript reflect the results of research conducted by the author(s) and do not necessarily reflect the official policy or position of the Uniformed Services University, Defense Health Agency, Department of Defense, nor the U.S. Government. Research data were derived from an approved MEDCoE Institutional Review Board protocol number (#25-00019n).

## Data availability

Data is available upon reasonable request and after satisfying any DHA data sharing agreements. Data sharing agreements applications can be found on health.mil.

## Funding

This project was supported in part by the Uniformed Services University, Department of Rehabilitation, Musculoskeletal Injury Rehabilitation Research for Operational Readiness (MIRROR) Program (HU00011920011). The funder had no role in the design, data collection, data analysis, data interpretation, or writing of the report.

## Declaration of interests

No authors have any conflicts to disclose.

## References

[1] Leifer V.P., Katz J.N., Losina E. (2022). The burden of OA-health services and economics. Osteoarthr. Cartil..

[2] Ibounig T., Simons T., Launonen A., Paavola M. (2020). Glenohumeral osteoarthritis: an overview of etiology and diagnostics. Scand. J. Surg..

[3] Ibounig T., Sanders S., Haas R. (2024). Systematic review of shoulder imaging abnormalities in asymptomatic adult shoulders (SCRUTINY): abnormalities of the glenohumeral joint. Osteoarthr. Cartil..

[4] Best M.J., Aziz K.T., Wilckens J.H., McFarland E.G., Srikumaran U. (2021). Increasing incidence of primary reverse and anatomic total shoulder arthroplasty in the United States. J. Shoulder Elb. Surg..

[5] Wagner E.R., Farley K.X., Higgins I., Wilson J.M., Daly C.A., Gottschalk M.B. (2020). The incidence of shoulder arthroplasty: rise and future projections compared with hip and knee arthroplasty. J. Shoulder Elb. Surg..

[6] Lowry V., Lavigne P., Zidarov D., Matifat E., Cormier A.A., Desmeules F. (2024). A systematic review of clinical practice guidelines on the diagnosis and management of various shoulder disorders. Arch. Phys. Med. Rehabil..

[7] Michener L.A., Heitzman J., Abbruzzese L.D. (2023). Physical therapist management of glenohumeral joint osteoarthritis: a clinical practice guideline from the American physical therapy association. Phys. Ther..

[8] Khazzam M., Gee A.O., Pearl M. (2020). Management of glenohumeral joint osteoarthritis. J. Am. Acad. Orthop. Surg..

[9] Galson S., Simon G. (2017). Real-World Evidence Generation and Evaluation of Therapeutics: Proceedings of a Workshop.

[10] Elliman T.D., Cohen B.S., Heaton K.J., Proctor S.P. (2022). Physical injuries, treatment-seeking, and perceived barriers to treatment in U.S. army drill sergeants. Mil. Med..

[11] Smith L., Westrick R., Sauers S. (2016). Underreporting of musculoskeletal injuries in the US army: findings from an infantry brigade combat team survey study. Sport Health.

[12] Sharp M.L., Fear N.T., Rona R.J. (2015). Stigma as a barrier to seeking health care among military personnel with mental health problems. Epidemiol. Rev..

[13] Pieters L., Lewis J., Kuppens K. (2020). An update of systematic reviews examining the effectiveness of conservative physical therapy interventions for subacromial shoulder pain. J. Orthop. Sports Phys. Ther..

[14] Cameron K.L., Driban J.B., Svoboda S.J. (2016). Osteoarthritis and the tactical athlete: a systematic review. J. Athl. Train..

[15] Benchimol E.I., Smeeth L., Guttmann A. (2015). The REporting of studies conducted using observational Routinely-collected health data (RECORD) statement. PLoS Med..

[16] Rhon D.I., Clewley D., Young J.L., Sissel C.D., Cook C.E. (2018). Leveraging healthcare utilization to explore outcomes from musculoskeletal disorders: methodology for defining relevant variables from a health services data repository. BMC Med. Inf. Decis. Mak..

[17] Rhon D.I., Greenlee T.A., Marchant B.G., Sissel C.D., Cook C.E. (2019). Comorbidities in the first 2 years after arthroscopic hip surgery: substantial increases in mental health disorders, chronic pain, substance abuse and cardiometabolic conditions. Br. J. Sports Med..

[18] George S.Z., Morton-Oswald S., Lee H., Horn M.E., Bhavsar N., Rhon D.I. (2024). IASP 2024 World Congress on Pain.

[19] Rhon D.I., Snodgrass S.J., Cleland J.A., Cook C.E. (2020). The risk of prior opioid exposure on future opioid use and comorbidities in individuals with non-acute musculoskeletal knee pain. J. Prim. Care Commun. Health.

[20] Ranganathan P., Pramesh C.S., Buyse M. (2015). Common pitfalls in statistical analysis: clinical versus statistical significance. Perspect Clin. Res..

[21] Bawa H.S., Weick J.W., Dirschl D.R. (2016). Gender disparities in osteoarthritis-related health care utilization before total knee arthroplasty. J. Arthroplast..

[22] Tschon M., Contartese D., Pagani S., Borsari V., Fini M. (2021). Gender and sex are key determinants in osteoarthritis not only confounding variables. A systematic review of clinical data. J. Clin. Med..

[23] Cameron K.L., Hsiao M.S., Owens B.D., Burks R., Svoboda S.J. (2011). Incidence of physician-diagnosed osteoarthritis among active duty United States military service members. Arthritis Rheum..

[24] Saltzman B.M., Leroux T.S., Verma N.N., Romeo A.A. (2018). Glenohumeral osteoarthritis in the young patient. J. Am. Acad. Orthop. Surg..

[25] Kong H., Wang X.Q., Zhang X.A. (2022). Exercise for osteoarthritis: a literature review of pathology and mechanism. Front. Aging Neurosci..

[26] Patzkowski J.C., Rivera J.C., Ficke J.R., Wenke J.C. (2012). The changing face of disability in the US army: the operation enduring freedom and operation Iraqi freedom effect. J. Am. Acad. Orthop. Surg..

[27] O’Sullivan O., Bennett A.N., Cameron K.L. (2024). Prevention of post-traumatic osteoarthritis in the military: relevance of OPTIKNEE and osteoarthritis action alliance recommendations. BMJ Mil. Health.

[28] Gorbaty J., Wally M.K., Odum S. (2023). Patients with glenohumeral arthritis are more likely to be prescribed opioids in the emergency department or urgent care setting. J. Opioid Manag..

[29] Anekar A.A., Hendrix J.M., Cascella M. (2025). StatPearls.

[30] Dowell D., Ragan K.R., Jones C.M., Baldwin G.T., Chou R. (2022). CDC clinical practice guideline for prescribing opioids for pain - united States, 2022. MMWR Recomm. Rep. (Morb. Mortal. Wkly. Rep.).

[31] Beard D.J., Rees J.L., Cook J.A. (2018). Arthroscopic subacromial decompression for subacromial shoulder pain (CSAW): a multicentre, pragmatic, parallel group, placebo-controlled, three-group, randomised surgical trial. Lancet.

[32] Ackerman I.N., Kemp J.L., Crossley K.M., Culvenor A.G., Hinman R.S. (2017). Hip and knee osteoarthritis affects younger people, too. J. Orthop. Sports Phys. Ther..

[33] Wilfong J.M., Badley E.M., Perruccio A.V. (2024). Old before their time? The impact of osteoarthritis on younger adults. Arthritis Care Res..

[34] Rhon D.I., Perez K.G., Eskridge S.L. (2019). Risk of post-traumatic knee osteoarthritis after knee injury in military service members. Muscoskel. Care.

