# Supplementary material for: Management of glenohumeral osteoarthritis in the younger patient: A population level analysis of usual care delivery in a large health system
Source: Osteoarthr Cartil Open. 2025 Nov 13;8(1):100705. doi: 10.1016/j.ocarto.2025.100705 (PMC12671371; doi:10.1016/j.ocarto.2025.100705)
Supplement: Multimedia component 1 [file mmc1.pdf]

Supplementary Appendix

Page	Item	
2	Figure SA1.	Non-pharmacological care distribution (Venn diagram)
3-4	Table SA1.	Characteristics of glenohumeral OA cohort compared by sex
5-6	Table SA2.	Pharmacological therapy compared by sex
6	Table SA3.	Non-pharmacological care by sex
7	Table SA4.	Imaging and surgical procedures by sex
8	Table SA5.	Shoulder diagnosis (IC9 and ICD10) codes
8-14	Table SA6.	Procedures based on Current Procedural Terminology (CPT) codes

Figure SA1. Non-pharmacological care distribution

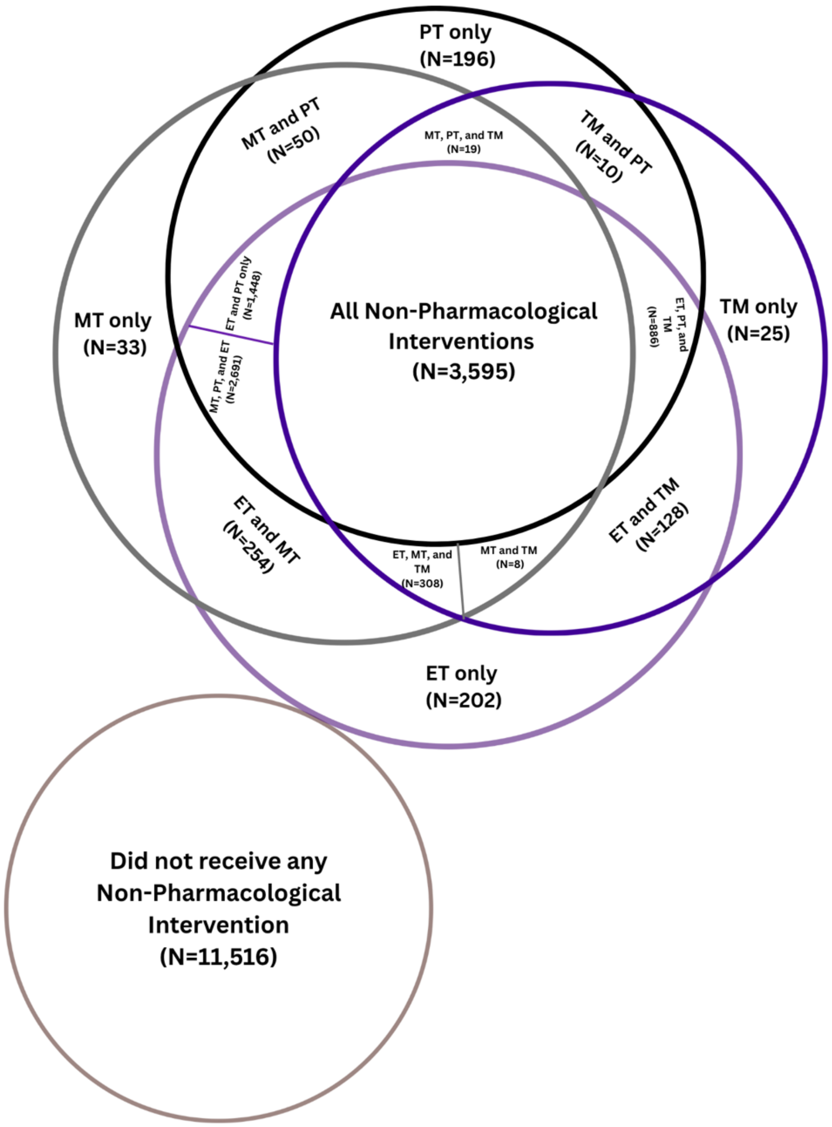

N = 11,516 (54.0%) did not receive either of these 3 interventions
ET = exercise therapy; PT = physiotherapy; MT = manual therapy; TM = therapeutic modalites

Table SA1. Characteristics of glenohumeral OA cohort compared by sex

	Total N=21,369	Female N=6,286 (29.4%)	Male N=12,374 (57.9%)	Missing N=2,709 (12.7%)
**Index Clinic Setting – (N%)**				
Private Sector	14,678 (68.7%)	4,915 (78.2%)	7,976 (64.5%)	1,787 (66.0%)
Military Treatment Facility	6,691 (31.3%)	1,371 (21.8%)	4,398 (35.5%)	922 (34.0%)
**Age**				
Mean (SD)	50.3 (9.9)	52.9 (8.6)	48.9 (10.3)	50.8 (10.0)
Median (Q1, Q3)	52.0 (44.0, 58.0)	54.0 (48.0, 60.0)	50.0 (42.0, 57.0)	53.0 (44.0, 59.0)
Range	18-66	18-66	18-66	18-66
**Beneficiary Category – (N%)**				
Active Duty	5,158 (24.1%)	364 (5.8%)	4,216 (34.1%)	578 (21.3%)
Dependent	5,788 (27.1%)	5,211 (82.9%)	465 (3.8%)	112 (4.1%)
Retired Service Member	8,710 (40.8%)	658 (10.5%)	7,048 (57.0%)	1,004 (37.1%)
Other	1,022 (4.8%)	4 (0.1%)	3 (0.02%)	1,015 (37.5%)
Inactive Guard/Reserve	691 (3.2%)	49 (0.8%)	642 (5.2%)	-
**Rank Status† – (N%)**				
Enlisted	16,133 (75.6%)	4,867 (77.4%)	9,269 (74.9%)	1,997 (73.7%)
Officer	5,193 (24.3%)	1,414 (22.5%)	3,098 (25.0%)	681 (25.1%)
Cadet	8 (0.04%)	-	7 (0.1%)	1 (0.04%)
Other/Unknown	20 (0.1%)	4 (0.1%)	-	16 (0.6%)
**Service Branch† – (N%)**				
Army	8,986 (42.1%)	2,592 (41.2%)	5,259 (42.5%)	1,135 (41.9%)
Air Force	6,060 (28.4%)	1,962 (31.2%)	3,325 (26.9%)	773 (28.5%)
Navy	4,118 (19.3%)	1,200 (19.1%)	2,399 (19.4%)	519 (19.2%)
Marines	1,515 (7.1%)	356 (5.7%)	979 (7.9%)	180 (6.6%)
Coast Guard	578 (2.7%)	137 (2.2%)	363 (2.9%)	78 (2.9%)
Other/Unknown	112 (0.5%)	39 (0.6%)	49 (0.4%)	24 (0.9%)
**Shoulder encounters per patient within 12 months before index visit**				
Mean (SD)	1.9 (3.0)	2.0 (3.2)	2.0 (3.0)	1.8 (2.9)
Median (Q1, Q3)	1 (0, 2)	1 (0, 2)	1 (0, 2)	1 (0, 2)
Range	0-58	0-35	0-58	0-43
**Shoulder encounters per patient within 12 months after index visit**				
Mean (SD)	7.9 (12.0)	9.8 (12.6)	9.5 (12.5)	8.5 (12.3)

Median (Q1, Q3)	3 (2, 7)	4 (2, 13)	4 (2, 13)	3 (1, 11)
Range	(0-76)	(0-105)	(0-112)	(0-123)
**Comorbidities at index visit**				
Insomnia	1,383 (6.5%)	461 (7.3%)	720 (5.8%)	202 (7.5%)
Sleep Apnea	3,067 (14.4%)	562 (8.9%)	2,088 (16.9%)	417 (15.4%)
Other Sleep Disorders	1,119 (5.2%)	263 (4.2%)	688 (5.6%)	168 (6.2%)
Depression	1,700 (8.0%)	809 (12.9%)	674 (5.4%)	217 (8.0%)
Anxiety	1,645 (7.7%)	762 (12.1%)	637 (5.1%)	246 (9.1%)
PTSD	693 (3.2%)	110 (1.7%)	488 (3.9%)	95 (3.5%)
Other Mental Health Disorders	1,622 (7.6%)	483 (7.7%)	895 (7.2%)	244 (9.0%)
Cardiovascular Disease	7,295 (34.1%)	2,356 (37.5%)	3,970 (32.1%)	969 (35.8%)
Metabolic Syndromes	8,188 (38.3%)	2,690 (42.8%)	4,430 (35.8%)	1,068 (39.4%)

Table SA2. Pharmacological therapy compared by sex

	Total N=21,369	Female N=6,286 (29.4%)	Male N=12,374 (57.9%)	Missing N=2,709 (12.7%)
**NSAID – (N%)**	3,154 (14.8%)	893 (14.2%)	1,871 (15.1%)	390 (14.4%)
Mean Unique Fills (SD)	8.5 (5.4)	8.5 (5.5)	8.5 (5.4)	8.8 (5.4)
Median Unique Fills (Q1, Q3)	8 (4, 12)	7 (4, 12)	7 (4, 12)	8 (5, 12)
Range	1-36	1-29	1-36	1-34
Median (Q1, Q3) days to first fill	19 (1, 60)	18 (0,57)	18 (1, 61)	21 (3, 61)
**Opioids – (N%)**	2,860 (13.4%)	810 (12.9%)	1,693 (13.7%)	357 (13.2%)
Mean Unique Fills (SD)	8.1 (8.9)	8.2 (8.7)	7.8 (8.7)	8.9 (10.5)
Median Unique Fills (Q1, Q3)	5 (2, 10)	5 (2, 11)	5 (2, 10)	5 (2, 10)
Range	1-86	1-56	1-86	1-61
Median (Q1, Q3) days to first fill	29 (7, 85)	28 (7, 85)	28 (7, 84)	35 (9, 90)
**Analgesics – (N%)**	1,926 (9.0%)	536 (8.5%)	1,162 (9.4%)	228 (8.4%)
Mean Unique Fills (SD)	2.4 (2.0)	2.3 (1.9)	2.4 (2.1)	2.5 (1.8)
Median Unique Fills (Q1, Q3)	2 (1, 3)	2 (1, 3)	2 (1, 3)	2 (1, 3)
Range	1-23	1-16	1-23	1-13
Median (Q1, Q3) days to first fill	92 (31, 190)	87.5 (30, 181)	94 (31, 196)	88.5 (33, 168.5)
**Muscle Relaxers – (N%)**	2,511 (11.8%)	713 (11.3%)	1,482 (12.0%)	316 (11.7%)
Mean Unique Fills (SD)	3.9 (3.6)	3.8 (3.6)	3.9 (3.6)	4.1 (3.8)
Median Unique Fills (Q1, Q3)	3 (1, 5)	3 (1, 5)	3 (1, 5)	3 (2, 6)
Range	1-33	1-27	1-29	1-33
Median (Q1, Q3) days to first fill	52 (8, 139)	47 (5, 123)	55.5 (9, 151)	49.5 (10, 123)
**Any Injections* – (N%)**	5,606 (26.2%)	1,783 (28.4%)	3,077 (24.9%)	746 (27.5%)
Mean number of injections (SD)	1.4 (0.7)	1.4 (0.8)	1.4 (0.7)	1.4 (0.7)
Median number of injections (Q1, Q3)	1 (1, 2)	1 (1, 1)	1 (1, 2)	1 (1, 2)
Range	1-14	1-8	1-14	1-6
Median (Q1, Q3) days to first	16 (0, 64)	17 (0, 69)	16 (0, 63)	15 (0, 63)
**Corticosteroid Injections – (N%)**	5,577 (26.1%)	1,773 (28.2%)	3,059 (24.7%)	745 (27.5%)
Mean number of injections (SD)	1.4 (0.7)	1.4 (0.8)	1.3 (0.7)	1.4 (0.7)
Median number of injections (Q1, Q3)	1 (1, 2)	1 (1, 2)	1 (1, 2)	1 (1, 2)
Range	1-14	1-8	1-14	1-6
Median (Q1, Q3) days to first	16 (0, 64)	1 (0, 69)	15 (0, 63)	15 (0, 63)
**Viscosuplementation Injections – (N%)**	57 (0.3%)	17 (0.3%)	34 (0.3%)	6 (0.2%)

Mean number of injections (SD)	1.3 (0.7)	1.4 (0.9)	1.3 (0.6)	1.3 (0.8)
Median number of injections (Q1, Q3)	1 (1, 1)	1 (1, 1)	1 (1, 1)	1 (1, 1)
Range	1-4	1-4	1-3	1-3
Median (Q1, Q3) days to first	56 (14, 136)	43 (10, 111)	101.5 (19, 154)	41 (15, 63)

Abbreviations: SD= Standard Deviation; Q1= 1st quartile; Q3= 3rd quartile; *=Includes any of corticosteroid or viscosupplementation injections.

Table SA3. Non-pharmacological care by sex

	Total N=21,369	Female N=6,286 (29.4%)	Male N=12,374 (57.9%)	Missing N=2,709 (12.7%)
**Physical Therapy – N (%)**	8,895 (41.6%)	2,638 (42.0%)	5,221 (42.2%)	1,036 (38.2%)
Mean (SD)	3.9 (7.1)	2.9 (5.6)	4.3 (7.6)	4.0 (7.6)
Median (Q1, Q3)	1 (1, 3)	1 (1, 2)	1 (1, 3)	1 (1, 3)
Range	(1-107)	(1-95)	(1-107)	(1-57)
Median (Q1, Q3) days to first procedure	35 (14, 85)	34 (14, 80)	35 (14, 87)	38 (14, 91.5)
**Passive Modalities – N (%)**	4,979 (23.3%)	1,590 (25.3%)	2,865 (23.2%)	524 (19.3%)
Mean (SD)	8.9 (9.7)	9.1 (9.7)	8.8 (9.7)	8.7 (9.4)
Median (Q1, Q3)	6 (2, 12)	6 (2, 12)	5 (2, 12)	5 (2, 11)
Range	(1-83)	(1-73)	(1-83)	(1-52)
Median (Q1, Q3) days to first procedure	42 (16, 101)	38.5 (16, 87)	44 (16, 106)	47 (17.5, 113)
**Exercise Therapy – N (%)**	9,512 (44.5%)	311 (4.9%)	714 (5.8%)	152 (5.6%)
Mean (SD)	11.0 (11.3)	11.4 (11.2)	10.9 (11.3)	10.8 (11.8)
Median (Q1, Q3)	7 (3, 15)	8 (3, 16)	7 (3, 15)	6 (2, 15)
Range	(1-106)	(1-73)	(1-105)	(1-106)
Median (Q1, Q3) days to first procedure	34 (13, 84)	31.5 (13, 78)	35 (13, 86)	34 (12, 87)
**Manual Therapy – N (%)**	6,958 (32.6%)	2,244 (35.7%)	3,922 (31.7%)	792 (29.2%)
Mean (SD)	9.8 (10.4)	10.4 (10.6)	9.5 (10.2)	9.5 (10.8)
Median (Q1, Q3)	6 (2, 13)	7 (3, 14)	6 (2, 13)	6 (2, 13)
Range	(1-109)	(1-84)	(1-76)	(1-109)
Median (Q1, Q3) days to first procedure	44 (17, 98)	39 (15, 86)	48 (19, 104)	47 (17, 107)
**Acupuncture – N (%)**	56 (0.3%)	12 (0.2%)	32 (0.3%)	12 (0.4%)
Mean (SD)	2.7 (5.5)	1.7 (1.1)	3.1 (7.1)	2.5 (2.5)
Median (Q1, Q3)	1 (1, 2)	1 (1, 2.5)	1 (1, 2)	1 (1, 3.5)
Range	1-41	1-4	1-41	1-9
Median (Q1, Q3) days to first procedure	97 (17, 197)	123.5 (30, 219)	104.5 (9, 193.5)	93 (29.5, 170)

Table SA4. Imaging and surgical procedures by sex

	Total N=21,369	Female N=6,286 (29.4%)	Male N=12,374 (57.9%)	Missing N=2,709 (12.7%)
**Radiographs – N (%)**	10,967 (51.3%)	3,523 (56.0%)	6,159 (49.8%)	1,285 (47.4%)
Mean (SD)	1.9 (1.4)	1.9 (1.4)	1.9 (1.4)	1.9 (1.4)
Median (Q1, Q3)	1 (1, 2)	1 (2, 2)	1 (1, 2)	1 (1, 2)
Range	(1-19)	(1-19)	(1-14)	(1-13)
Median (Q1, Q3) days to first radiograph	0 (0, 17)	0 (0, 12)	0 (0, 18)	0 (0, 28)
**Advanced imaging (MRIs, arthrograms, CT scans) - N (%)¥**	10,068 (47.1%)	2,997 (47.7%)	5,898 (47.7%)	1,173 (43.3%)
Mean (SD)	1.8 (0.9)	1.8 (0.9)	1.9 (0.9)	1.8 (0.9)
Median (Q1, Q3)	2 (1, 2)	2 (1, 2)	2 (1, 2)	2 (1, 2)
Range	(1-10)	(1-10)	(1-10)	(1-6)
Median (Q1, Q3) days to first scan	0 (0, 22)	0 (0, 23)	0 (0, 20)	0 (0, 32)
**Any imaging procedure – N (%)**	15,313 (71.7%)	4,862 (77.3%)	8,654 (69.9%)	1,797 (66.3%)
Mean (SD)	2.5 (1.8)	2.4 (1.8)	2.5 (1.9)	2.5 (1.8)
Median (Q1, Q3)	2 (1, 3)	2 (1, 3)	2 (1, 3)	2 (1, 3)
Range	1-27	(1-27)	(1-20)	(1-16)
Median (Q1, Q3) days to first procedure	0 (0, 0)	0 (0, 0)	0 (0, 0)	0 (0,0)
**Surgery – N (%)**				
Shoulder Arthroscopy	4,031 (18.9%)	993 (15.8%)	2,585 (20.9%)	453 (2.1%)
- Mean (SD) days to arthroscopy	91.2 (92.1)	89.7 (93.5)	90.1 (90.6)	100.8 (97.1)
Shoulder Arthroplasty	41 (0.2%)	6 (0.1%)	28 (0.2%)	7 (0.3%)
- Mean (SD) days to arthroplasty	130.4 (98.2)	45.2 (43.5)	146.0 (104.5)	141.1 (71.5)
Other Surgical Shoulder Procedure†	1,177 (5.5%)	215 (3.4%)	822 (6.6%)	140 (5.2%)
- Mean (SD) days to other procedure	100.5 (96.3)	103.0 (99.8)	95.8 (93.2)	124.8 (105.7)

Abbreviations: SD= Standard Deviation; Q1= 1st quartile; Q3= 3rd quartile ; MRI = magnetic resonance imaging ; CT = computed tomography.

Active duty refers to individuals actively serving in the military full time, in comparison to any individual not in military service (either a dependent or someone separated or retired from military service).

¥ The majority of individuals who received advanced imaging procedures had an MRI (98.2% total, 98.1% female, 98.3% male, 98.5% missing)

† Examples of other shoulder procedures included capsular release, open Bankart repair, biceps tenodesis

Table SA5. Shoulder Diagnosis (IC9 and ICD10) Codes

ICD10 Code WITHOUT Decimals	ICD10 Code WITH decimals	Description
M19011	M19.011	Primary osteoarthritis, right shoulder
M19012	M19.012	Primary osteoarthritis, left shoulder
M19019	M19.019	Primary osteoarthritis, unspecified shoulder
M19111	M19.111	Post-traumatic osteoarthritis, right shoulder
M19112	M19.112	Post-traumatic osteoarthritis, left shoulder
M19119	M19.119	Post-traumatic osteoarthritis, unspecified shoulder
M19211	M19.211	Traumatic arthropathy, right shoulder
M19212	M19.212	Traumatic arthropathy, left shoulder
M19219	M19.219	Traumatic arthropathy, unspecified shoulder
M1990	M19.90	Unspecified osteoarthritis, unspecified shoulder
M1991	M19.91	Unspecified osteoarthritis, right shoulder
M1992	M19.92	Unspecified osteoarthritis, left shoulder
**ICD-9 Code WITHOUT decimals**	**ICD-9 Code WITH decimals**	**Description**
71511	715.11	Osteoarthrosis, localized, primary, shoulder region
71521	715.21	Osteoarthrosis, localized, secondary, shoulder region
71531	715.31	Osteoarthrosis, localized, not specified whether primary or secondary, shoulder region
71591	715.91	Osteoarthrosis, unspecified whether generalized or localized, shoulder region

Table SA6. Procedures based on Current Procedural Terminology (CPT) and Healthcare Common Procedure Coding System (HCPCS) codes

Procedure Codes	Description
**Pharmaceutical Injection**	
20526	Injection, therapeutic (eg, local anesthetic, corticosteroid), carpal tunnel Carpal tunnel
20527	Injection, enzyme (eg, collagenase), palmar fascial cord (ie, Dupuytren's contracture)
20550	Injection(s); single tendon sheath, or ligament, aponeurosis (eg, plantar “fascia”) Tendon Sheath
20551	Injection(s); single tendon origin/insertion Tendon Sheath
20552	Injection(s); single or multiple trigger point(s), one or two muscle(s) Muscle
20553	Injection(s); single or multiple trigger point(s) three or more muscle(s) Muscle
20600	Arthrocentesis, aspiration and/or injection, small joint or bursa (eg, fingers, toes); without ultrasound guidance
20604	Arthrocentesis, aspiration and/or injection, small joint or bursa (eg, fingers, toes); with ultrasound guidance, with permanent recording and reporting
20605	Arthrocentesis, aspiration and/or injection, intermediate joint or bursa (eg, temporomandibular, acromioclavicular, wrist, elbow or ankle, olecranon bursa); without ultrasound guidance
20606	Arthrocentesis, aspiration and/or injection, intermediate joint or bursa (eg, temporomandibular, acromioclavicular, wrist, elbow or ankle, olecranon bursa); with ultrasound guidance, with permanent recording and reporting

20610	Arthrocentesis, aspiration and/or injection, major joint or bursa (eg, shoulder, hip, knee, subacromial bursa) without ultrasound guidance
20611	Arthrocentesis, aspiration and/or injection, major joint or bursa (eg, shoulder, hip, knee, subacromial bursa) with ultrasound guidance
20612	Aspiration and/or injection of ganglion cyst(s) any location
20615	Aspiration and injection for treatment of bone cyst
28899	Unlisted procedure, foot or toes; Note: Use 28899 for tarsal tunnel injection.** (this is senstivit, but not specific - don't use this code for most cases)
J3490	Unclassified injectible prescription drugs
**Viscosupplementation/ Hyaluronic Acid Injection**	
J7318	Hyaluronan or derivative, durolane, for intra-articular injection, 1 mg
J7320	Hyaluronan or derivitive, genvisc 850, for intra-articular injection, 1 mg
J7321	Hyaluronan or derivative, hyalgan, supartz or visco-3, for intra-articular injection, per dose
J7322	Hyaluronan or derivative, hymovis, for intra-articular injection, 1 mg
J7323	Hyaluronan or derivative, euflexxa, for intra-articular injection, per dose
J7324	Hyaluronan or derivative, orthovisc, for intra-articular injection, per dose
J7325	Hyaluronan or derivative, synvisc or synvisc-one, for intra-articular injection, 1 mg
J7326	Hyaluronan or derivative, gel-one, for intra-articular injection, per dose
J7327	Hyaluronan or derivative, monovisc, for intra-articular injection, per dose
J7328	Hyaluronan or derivative, gelsyn-3, for intra-articular injection, 0.1 mg
J7329	Hyaluronan or derivative, trivisc, for intra-articular injection, 1 mg
J7331	Hyaluronan or derivative, synojoynt, for intra-articular injection, 1 mg
J7332	Hyaluronan or derivative, triluron, for intra-articular injection, 1 mg
**Corticosteroid Injections**	
J1010	Injection, methylprednisolone acetate, 1 mg
J1020	Injection, methylprednisolone acetate
J1030	Injection, methylprednisolone acetate
J1040	Injection, methylprednisolone acetate
J1094	Injection, dexamethasone acetate, 1 mg
J0702	Injection, betamethasone acetate 3 mg and betamethasone sodium phosphate 3 mg
J1100	Injection, dexamethasone sodium phosphate, 1 mg
J1700	Injection, hydrocortisone sodium phosphate
J1710	Injection, hydrocortisone sodium phosphate, up to 50 mg
J1720	Injection, hydrocortisone sodium succinate, up to 100 mg
J2930	Methylprednisolone Sodium Succinate

J3300	Injection, triamcinolone acetonide, preservative free, 1 mg
J3301	Injection, triamcinolone acetonide, not otherwise specified, 10 mg
J3302	Injection, triamcinolone diacetate, per 5 mg
J3303	Injection, triamcinolone diacetate, per 5 mg
**Physical Therapy**	
97001	Physical therapy evaluation (UNTIMED CODE)
97002	Physical therapy re-evaluation (UNTIMED CODE)
97161	Physical therapy evaluation; low complexity: typically 20 min face to face time
97162	Physical therapy evaluation; moderate complexity: typically 30 min face to face time
97163	Physical therapy evaluation; moderate complexity: typically 45 min face to face time
97164	Re-evaluation of physical therapy established plan of care; Typically 20 min spent face to face with patient and/or family
97002	Physical therapy re-evaluation
V571*	Care involving other physical therapy
**Therapeutic Modalities**	
97014	Application of modality to one or more areas; electrical stimulation (unattended)
97032	Application of a modality to one or more areas; electrical stimulation (manual)
G0283	Electrical stimulation other than wound
97033	Application of a modality to one or more areas; iontophoresis, each 15 minutes
97026	Application of a modality to one or more areas; infrared
97035	Application of a modality to one or more areas; ultrasound, each 15 minutes
97028	Application of a modality to one or more areas; ultraviolet
97010	Application of modality to one or more area; hot or cold packs
97016	Application of a modality to one or more areas; vasopneumatic devices
**Exercise/Active Therapy**	
97110	Therapeutic procedure, one or more areas, each 15 minutes; therapeutic exercises to develop strength and endurance, range of motion and flexibility
97112	Therapeutic procedure, one or more areas, each 15 minutes; neuromuscular reeducation of movement, balance, coordination, kinesthetic sense, posture, and/or proprioception for sitting and/or standing activities
97113	Therapeutic procedure, one or more areas, each 15 minutes; aquatic therapy with therapeutic exercise
97116	Therapeutic procedure, one or more areas, each 15 minutes; gait training (includes stair climbing)
97150	Therapeutic procedure(s), group (2 or more individuals) 97530 Therapeutic activities, direct (one-on-one) patient contact by the provider (use of dynamic activities to improve functional performance), each 15 minutes

97530	Therapeutic activities, direct (one-on-one) patient contact by the provider (use of dynamic activities to improve functional performance), each 15 minutes
97537	Community/work reintegration training (eg, shopping, transportation, money management, avocational activities and/or work environment/modification analysis, work task analysis, use of assistive technology device/adaptive equipment), direct one-on-one contact, each 15 minutes
97542	wheel chair management
97545	Work hardening/conditioning, initial 2 hours
97546	Work hardening/conditioning, each additional hour
97750	Physical performance test
**Manual Therapy**	
97140	Manual therapy techniques (e.g., mobilization/manipulation, manual lymphatic draining, manual traction), one or more regions, each 15 minutes
98925	Osteopathic Manipulation
98926	Osteopathic Manipulation
98927	Osteopathic Manipulation
98928	Osteopathic Manipulation
98929	Osteopathic Manipulation
98940	Chiropractic manipulative treatment (CMT); spinal, one to two regions
98941	Chiropractic manipulative treatment (CMT); spinal, three or four regions
98942	Chiropractic manipulative treatment (CMT); spinal, five regions
98943	Chiro, manipulation, extraspinal, one or more regions
**Acupuncture**	
97780	acupuncture, one or more needles; without electrical stimulation
97781	acupuncture, one or more needles; with electrical stimulation
97810	Acupuncture, one or more needles; without electrical stimulation, initial 15 minutes of personal one-on- one contact with the patient
97811	Acupuncture, one or more needles; without electrical stimulation, each additional 15 minutes of personal one-on-one contact with the patient, with reinsertion of needle(s) (list separately in addition to code for primary procedure)
97813	Acupuncture, one or more needles; with electrical stimulation, initial 15 minutes of personal one-on-one contact with the patient
97814	Acupuncture, one or more needles; with electrical stimulation, each additional 15 minutes of personal one- on-one contact with the patient, with reinsertion of needle(s) (list separately in addition to code for primary procedure)
S8930	Electrical Stimulation of auricular Acupunture point; each 15 minutes of personal one-on-one cotact with patient
**Radiographs**	
73000	Radiologic examination, clavicle, complete
73010	Radiologic examination, scapula, complete
73020	Radiologic examination, shoulder; 1 view

73030	Radiologic examination, shoulder; complete, minimum of 2 views
73040	Radiologic examination, shoulder, arthrography, radiological supervision and interpretation
73050	Radiologic examination; acromioclavicular joints, bilateral, with or without weighted distraction
73060	Radiologic examination; humerus, minimum of 2 views
**MRI**	
73218	MRI, upper extremity, other than joint; without contrast material(s)
73219	MRI, upper extremity, other than joint; with contrast material(s)
73220	MRI, upper extremity, other than joint; without contrast material(s), followed by contrast material(s) and further sequences
73221	MRI, any joint of upper extremity; without contrast material(s)
73222	MRI, any joint of upper extremity; with contrast material(s)
73223	MRI, any joint of upper extremity; without contrast material(s), followed by contrast material(s) and further sequences
**CT**	
73200	CT, upper extremity, without contrast material
73201	CT, upper extremity, with contrast material(s)
73202	CT, upper extremity, without contrast material, followed by contrast material(s) and further sections
**Arthrogram**	
73040	Radiologic examination, shoulder, arthrography, radiological supervision and interpretation
**Shoulder Arthroscopy**	
29805	Arthroscopy, shoulder, diagnostic, with or without synovial biopsy (separate procedure)
29806	Arthroscopy, shoulder, surgical; capsulorrhaphy
29807	Arthroscopy, shoulder, surgical; repair of SLAP lesion
29815	Arthroscopy, shoulder, diagnostic, with or without synovial biopsy [separate procedure]
29819	Arthroscopy, shoulder, surgical; with removal of loose body or foreign body
29820	Arthroscopy, shoulder, surgical; synovectomy, partial
29821	Arthroscopy, shoulder, surgical; synovectomy, complete
29822	Arthroscopy, shoulder, surgical; debridement, limited
29823	Arthroscopy, shoulder, surgical; debridement, extensive
29824	Arthroscopy, shoulder, surgical; distal claviculectomy including distal articular surface
29825	Arthroscopy, shoulder, surgical; with lysis and resection of adhesions, with or without manipulation
29826	Arthroscopy, shoulder, surgical; decompression of subacromial space with partial acromioplasty, with coracoacromial ligament release, when performed
29827	Arthroscopy, shoulder, surgical; with rotator cuff repair
29828	Treatment for arthroscopic surgical biceps tenodesis

S2300	Arthroscopy, shoulder, surgical; with thermally-induced capsulorrhaphy
**Shoulder Arthroplasty**	
23470	Arthroplasty, GH joint; hemiarthroplasty
23472	Arthroplasty, GH joint; total shoulder (glenoid and proximal humeral replacement)
23473	Revision of total shoulder arthroplasty, including allograft when performed; humeral or glenoid component
23474	Revision of total shoulder arthroplasty, including allograft when performed; humeral and glenoid component
**Other Shoulder Surgery**	
23395	Muscle transfer, any type, shoulder or upper arm; single
23397	Muscle transfer, any type, shoulder or upper arm; multiple
23400	Scapulopexy (eg, Sprengels deformity or for paralysis)
23405	Tenotomy, shoulder area; single tendon
23406	Tenotomy, shoulder area; multiple tendons through same incision
23410	Repair of ruptured musculotendinous cuff open; acute
23412	Repair of ruptured musculotendinous cuff open; chronic
23415	Coracoacromial ligament release, with or without acromioplasty
23420	Reconstruction of complete shoulder cuff avulsion, chronic (includes acromioplasty)
23430	Tenodesis of long tendon of biceps
23440	Resection or transplantation of long tendon of biceps
23450	Capsulorrhaphy, anterior, Putti-Platt procedure or Magnuson type operation
23455	Capsulorrhaphy, anterior, Putti-Platt procedure or Magnuson type operation with labral repair
23460	Capsulorrhaphy, anterior, any type; with bone block
23462	Capsulorrhaphy, anterior, any type; with coracoid process transfer
23465	Capsulorrhaphy, GH joint, posterior, with or without bone block
23466	Capsulorrhaphy, GH joint, any type multi-directional instability
23480	Osteotomy, clavicle, with or without internal fixation
23485	Osteotomy, clavicle, with or without internal fixation; with bone graft for nonunion or malunion
23490	Prophylactic treatment (nailing, pinning, plating or wiring) with or without methyl methacrylate; clavicle
23491	Prophylactic treatment (nailing, pinning, plating or wiring) with or without methyl methacrylate; proximal humerus

Note: For non-imaging and non-specific procedure codes, they must have been present along with a shoulder diagnosis code in the same encounter to count.
